# DNA metabarcoding reveals introduced species predominate in the diet of a threatened endemic omnivore, Telfair’s skink (*Leiolopisma telfairii*)

**DOI:** 10.1002/ece3.8484

**Published:** 2021-12-21

**Authors:** Maximillian P. T. G. Tercel, Rosemary J. Moorhouse‐Gann, Jordan P. Cuff, Lorna E. Drake, Nik C. Cole, Martine Goder, Rouben Mootoocurpen, William O. C. Symondson

**Affiliations:** ^1^ School of Biosciences Cardiff University Cardiff UK; ^2^ Durrell Wildlife Conservation Trust Trinity Jersey; ^3^ Department of Animal & Plant Sciences NERC Biomolecular Analysis Facility Sheffield UK; ^4^ Rothamsted Insect Survey, Rothamsted Research Harpenden UK; ^5^ Mauritian Wildlife Foundation Vacoas Mauritius

**Keywords:** dietary analysis, invasive species, island restoration, multiple markers, *Pheidole megacephala*, reptiles, Round Island Mauritius

## Abstract

Introduced species can exert disproportionately negative effects on island ecosystems, but their potential role as food for native consumers is poorly studied. Telfair's skinks are endemic omnivores living on Round Island, Mauritius, a globally significant site of biodiversity conservation. We aimed to determine the dietary diversity and key trophic interactions of Telfair's skinks, whether introduced species are frequently consumed, and if diet composition changes seasonally between male and female skinks. We used DNA metabarcoding of skink fecal samples to identify animals (COI) and plants (ITS2) consumed by skinks. There were 389 dietary presence counts belonging to 77 dietary taxa found across the 73 Telfair's skink fecal samples. Introduced taxa were cumulatively consumed more frequently than other categories, accounting for 49.4% of all detections, compared to cryptogenic (20.6%), native (20.6%), and endemic taxa (9.5%). The most frequently consumed introduced species was the ant, *Pheidole megacephala*, present in 40% of samples. Blue latan palm, *Latania loddigesii*, was the most frequently consumed endemic species, present in 33% of samples but was only detected in the dry season, when fruits are produced. We found a strong seasonal difference in diet composition explained by the presence of certain plant species solely or primarily in one season and a marked increase in the consumption of animal prey in the dry season. Male and female skinks consumed several taxa at different frequencies. These results present a valuable perspective on the role of introduced species in the trophic network of their invaded ecosystem. Both native and introduced species provide nutritional resources for skinks, and this may have management implications in the context of species conservation and island restoration.

## Graphical abstract

Telfair's skinks are keystone omnivores found on Round Island, Mauritius. Using a multimarker DNA metabarcoding approach, we identify that this endemic species frequently consumes introduced species, which comprise almost 50% of all prey detections. This provides a valuable insight into how introduced species may fit into the food web in their introduced range.

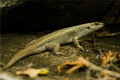

## 1 INTRODUCTION

A novel species introduced into a new ecosystem can interact with taxa already present in a variety of ecological roles, for example, as a mutualist (Kaiser‐Bunbury et al., 2011), competitor (Cole & Harris, 2011), predator (O’Dowd et al., 2003), prey (Li et al., 2011), or parasite (Arbetman et al., 2013). Introduced species are often associated with network and community restructuring (Memmott et al., 2000; Russo et al., 2014), as well as native biodiversity declines (Clavero & Garcia‐Berthou, 2005; Luque et al., 2014) and even ecological collapse (O’Dowd et al., 2003). However, introduced species may have more nuanced effects on ecosystems, including interactions beneficial to native species (Schlaepfer et al., 2011). For example, non‐native trees may provide nesting sites to threatened birds (Schlaepfer et al., 2011) and non‐native plants may provide floral resources to a range of threatened native pollinators (Baldock et al., 2015).

Introduced species have been studied extensively as invasive predators and herbivores, but their role in the diet of native species has been given less attention. A few studies examine this subject explicitly. For example, Ando et al. (2013) showed that the critically endangered red‐headed wood pigeon, *Columba janthina nitens*, consumed introduced plants more frequently than native species on the Ogasawara Islands, Japan. Similarly, introduced species were consumed frequently by the Ogasawara buzzard, *Buteo buteo oyoshim*, with 90% of its diet consisting of introduced animals (Kato & Suzuki, 2005). These small oceanic islands harbor high levels of endemism. Introduced species are typically associated with disproportionately negative effects on island biodiversity (Sax & Gaines, 2009), but are shown to provide nutritional resources to these endemic species. This may be more common than currently acknowledged, with introduced species representing a significant dietary element for native consumers.

**FIGURE 1:**
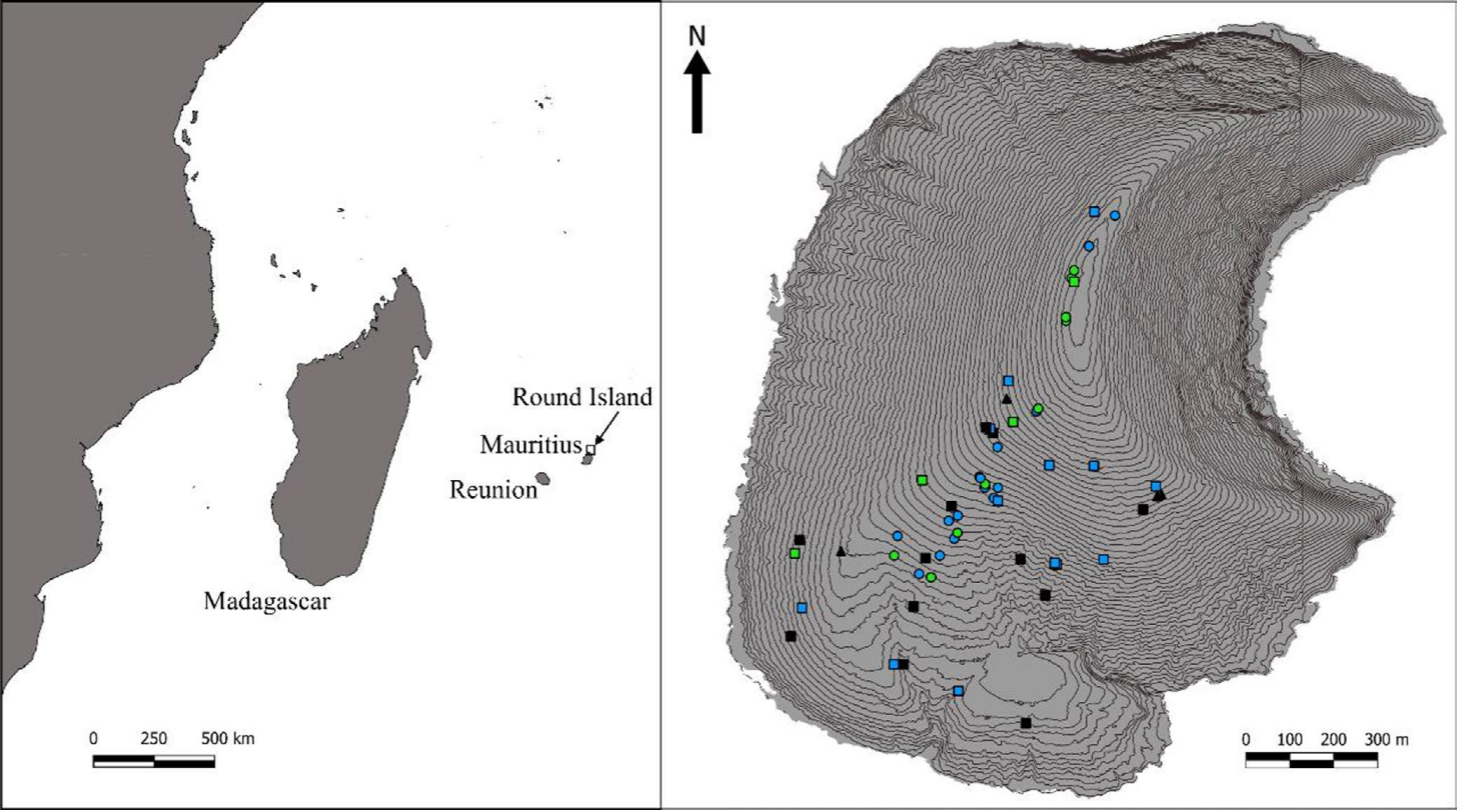
Location of the study. The left map shows the location of Round Island in the Indian Ocean. The right map shows the topography of Round Island (5 m contour lines) and the sampling locations for each skink. Symbol shape denotes the season samples were collected: wet = square, dry = circle, unknown = triangle. Symbol color denotes sex of the skinks samples were collected from: green = females, blue = males, black = unknown

**FIGURE 2:**
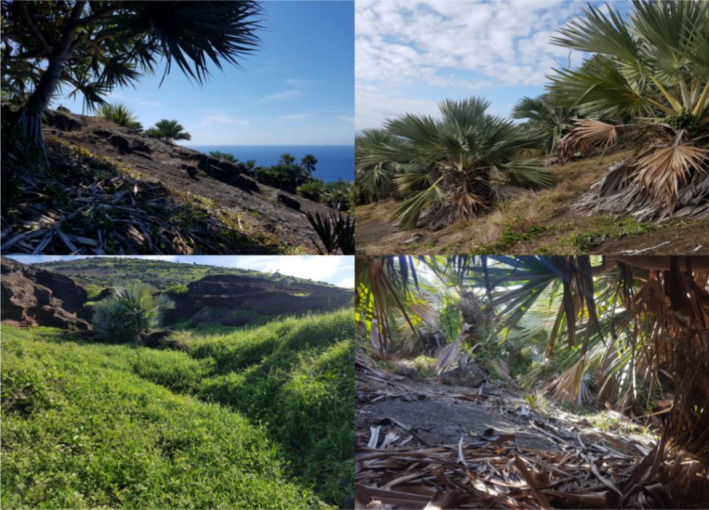
Habitats of Round Island. Top left: rock slab. These areas are scattered sparsely with *Pandanus vandermeschii* and *Latania loddigesii* trees and little other vegetation. Top right: regenerating palm forest. Primarily found on the Western and Northern parts of Round Island, these feature stands of one or a few *L*. *loddigesii* separated by herbaceous introduced and native plants. Bottom right: palmoid thicket habitat. Primarily closed canopy *L*. *loddigesii* trees. Bottom left: mixed weed habitat. This habitat consists of introduced and native herbaceous plants but may also feature stands of tightly packed regenerating native trees

**FIGURE 3:**
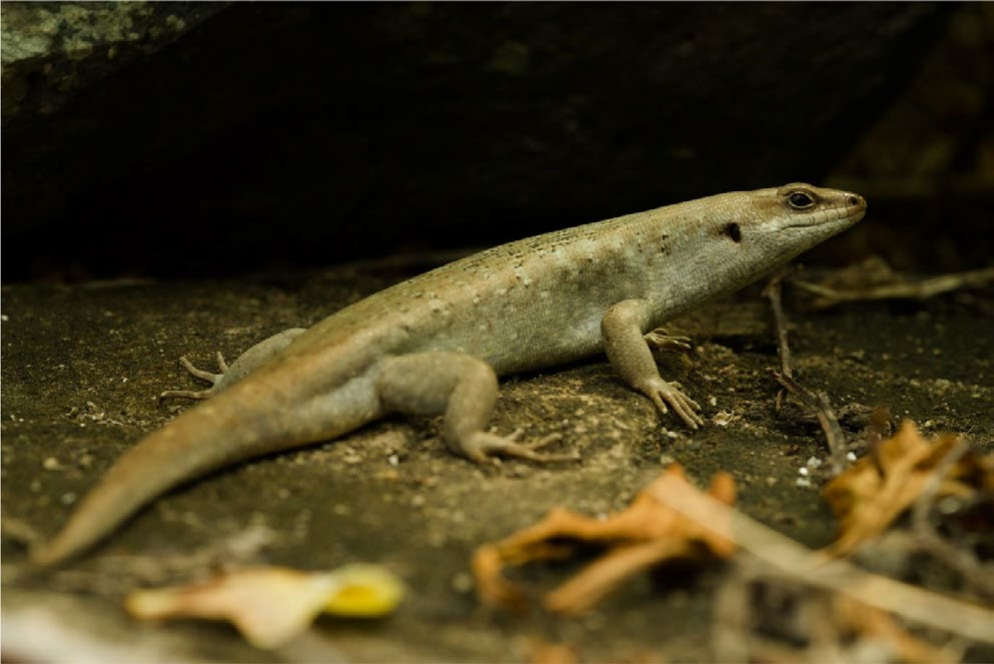
Telfair's skink, *Leiolopisma telfairii*

Round Island, situated 22.5 km North‐East of Mauritius (Figure 1), is a globally significant site of biodiversity conservation and now represents the last remnant of native lowland palm habitat (Figure 2) in the Mascarenes (Cheke & Hume, 2008). The palm habitat has been recovering since the eradication of goats, *Capra aegagrus hircus*, in 1979, and rabbits, *Oryctolagus cuniculus*, in 1986 (Cheke & Hume, 2008; Merton, 1987). At just 2.19 km^2^, it is home to several reptile species extirpated from mainland Mauritius by introduced species and habitat destruction. Telfair's skinks, *Leiolopisma telfairii* (Figure 3), are vulnerable omnivorous reptiles, typically growing to approximately 30 cm in total length, and are endemic to Mauritius. They became restricted to Round Island by the mid‐1800s because of the introduction of non‐native predators, such as rats (Cole, Goder, et al., 2018). The species has now been re‐introduced to the island Nature Reserves, Ile aux Aigrettes (0.26 km^2^, located 600 m from South‐East Mauritius), and Gunner's Quoin (0.7 km^2^, 5 km to the North of Mauritius) (Cole, Goder, et al., 2018). Round Island has been a designated nature reserve since 1957 and has never suffered from introduced terrestrial vertebrate predators, which have caused the extirpation and extinction of multiple Mauritian species elsewhere (Cheke & Hume, 2008). Habitat restoration efforts on Round Island since the 1980s have led to the recovery of its reptile populations, which includes seven species, four of which became restricted to the island by the mid‐19th century (Cole, Mootoocurpen, et al., 2018; North et al., 1994).

Previous dietary analyses of Telfair's skinks include morphological identification of food items and molecular analyses (Brown et al., 2014; Moorhouse‐Gann et al., 2021; Pernetta et al., 2005; Zuël, 2009). Morphological examination of feces shows that Telfair's skinks consume a variety of introduced and native species of fruit, seeds, arthropods, and vertebrates (Pernetta et al., 2005; Zuël, 2009). However, morphological methods of diet analysis can be unreliable and taxonomically imprecise, even when researchers are skilled. These methods also fail to adequately detect small or soft‐bodied prey (Pompanon et al., 2012; Symondson, 2002). Molecular approaches, especially those using high‐throughput sequencing (HTS), can provide much greater precision, frequently identifying taxa in fecal samples to species (Pompanon et al., 2012; Symondson, 2002; Taberlet et al., 2012). Previous HTS‐based fecal analysis of Telfair's skinks targeting plant (Moorhouse‐Gann et al., 2021) and animal (Brown et al., 2014) food resources on Ile aux Aigrettes and Round Island confirmed skinks eat a diverse range of taxa.

Identifying the diet of omnivores is challenging, but a few studies have facilitated the most comprehensive complex dietary assessments to date using DNA metabarcoding (Bonin et al., 2020; De Barba et al., 2014; Robeson et al., 2018; Silva et al., 2019). Trophic generalists may be central to ecological networks and can elicit top‐down effects across their entire breadth and depth. Deciphering the structure and dynamics of these interactions is therefore valuable, especially within a conservation context. Telfair's skinks are large, locally abundant trophic generalists endemic to Mauritius (Cole, Goder, et al., 2018; Jones, 1993; Vinson & Vinson, 1969), and are therefore likely to exert strong top‐down pressures on the ecological network of Round Island.

Here, we aimed to study the complete diet of Telfair's skinks on Round Island by using broad‐coverage plant and animal DNA metabarcoding primers. In doing so, we aimed to show: (a) the dietary diversity and key trophic interactions of Telfair's skinks; (b) whether introduced species feature prominently in the diet; (c) whether diet composition changes between seasons; and (d) whether diet composition is different between male and female skinks, which may have implications for conservation management and reintroduction initiatives.

## 2 METHODS

### 2.1 Study site

Round Island (Figure 1) is a basaltic cone that reaches 280 m above sea level and retains the last remnant of a native lowland palm habitat within the Mascarenes (Cheke & Hume, 2008), which has been recovering since introduced vertebrate herbivores were eradicated (Cheke & Hume, 2008; Merton, 1987). This habitat is primarily dominated by the blue latan palm, *Latania loddigesii*. Introduced herbaceous plants, such as *Achyranthes aspera* and *Tridax procumbens*, form swathes of invaded habitat in large open clearings between thickets of native trees. Before the 1980s, much of the island suffered deforestation caused by the introduced herbivores, resulting in the loss of all but two hardwood tree species represented by a single individual bois buis, *Fernelia buxifolia*, tree and a few individuals of acacia indigéne, *Gagnebina pterocarpa* (Strahm, 1993). Loss of habitat led to extensive soil erosion and created large expanses of barren rock slab over much of the island (Figure 2). Since 2002, there have been extensive efforts to restore the lost hardwood forests and to enhance the natural regeneration of the palm habitat (Jones, 2008). The invertebrate community is poorly studied, with few native species formally identified and described (Moldowan et al., 2016). Several introduced invertebrate species are now established over much of the island, such as the ants *Pheidole megacephala* and *Brachymyrmex cordemoyi*, and the webspinner *Oligotoma saundersii*. Endemic species of arthropod are common in their favored habitats, such as the Round Island stick insect, *Apterograeffea marshallae*, a herbivore of *L*. *loddigesii* (Moldowan et al., 2016), and the Serpent Island centipede, *Scolopendra abnormis*, a large invertebrate predator (Lewis et al., 2010). Many common invertebrates are yet to be described but are presumed to be native, such as a hyperabundant but undescribed cockroach species.

The vertebrate community consists of regionally important seabird colonies, a remnant endemic reptile assemblage and two introduced land bird species (Cheke & Hume, 2008; Cole, Mootoocurpen, et al., 2018). Seven endemic reptile species survive on Round Island because of the absence of introduced predators. Five of these are listed as Threatened on the IUCN Red List: Bojer's skink, *Gongylomorphus bojerii*, Durrell's Night gecko, *Nactus durrellorum*, keel‐scaled boa, *Casarea dussumieri*, Round Island day gecko, *Phelsuma guentheri*, and Telfair's skink, *Leiolopisma telfairii*, (IUCN, 2020). Additionally, two tortoise species, Aldabra giant tortoise, *Aldabrachelys gigantea*, and radiated tortoise, *Astrochelys radiata*, have been introduced to Round Island as “ecological replacements” for extinct Mauritian tortoises, *Cylindraspis* spp. (Griffiths et al., 2010). As the largest and one of the most abundant of the island's lizards, Telfair's skinks constitute the largest component of animal biomass of any omnivore on the island (Cole, Mootoocurpen, et al., 2018) and likely have a significant role within the island's food web dynamics.

Broad dry and wet seasons exist in Mauritius (Senapathi et al., 2010). The dry season typically begins in May and is characterized primarily by low rainfall, mean air temperature of ~20.5°C, and stronger winds, with the driest months being September and October. The wet season typically begins in December and is characterized by much more frequent rainfall, mean air temperature of ~24.5°C, and minimal winds, with the wettest months being January and February (Senapathi et al., 2010).

### 2.2 Skink sampling on Round Island

Fecal samples were collected in March, June, July, and December 2015 (Figure 1). Skinks were caught opportunistically by noose or hand after which defecation was induced using a gentle abdominal massage. The fecal samples were placed in polythene bags and dried over silica gel. Telfair's skinks are present over the entire island, but, unfortunately, some areas of the island are too dangerous to capture these fast‐moving reptiles. Skinks were released unharmed within ten minutes of capture at the locations where they were caught. Fecal samples were collected from 196 individual Telfair's skinks (identified by their sex, size that were recorded and distinguishing markings and body deformations, which were photographed) on Round Island and previously underwent DNA metabarcoding to identify the floral component of skink diet (Moorhouse‐Gann, 2018; Moorhouse‐Gann et al., 2021). Due to funding constraints, we were only able to advance 82 samples to sequencing, which were randomly selected for the current study from both dry (40) and wet (42) seasons.

### 2.3 Primer selection

Animal primers were tested in silico with a broad range of vertebrate and invertebrate taxa using PrimerMiner (Elbrecht & Leese, 2017) and in vitro with DNA extracted from animals sampled on Round Island. BerenF‐LuthienR (Cuff et al., 2020) provided the most comprehensive coverage, amplifying all Round Island invertebrate DNA extracts tested. UniPlant primer pair (Moorhouse‐Gann et al., 2018) was used to amplify the ITS2 DNA barcode in plants and successfully amplify almost all plant species found on Round Island.

### 2.4 DNA extraction, PCR amplification, and sequencing

DNA extraction from Telfair's skink fecal samples and preparation of plant DNA for 250‐bp paired‐end Illumina MiSeq high‐throughput sequencing followed Moorhouse‐Gann et al. (2021; Supplementary Information S2 and Table S1).

We used the following procedure to identify animal prey in the diet of Telfair's skinks. Polymerase chain reactions (PCRs) used 25 μl reaction volumes containing 5 μl DNA template, 12.5 μl of multiplex PCR mix (Qiagen, Manchester, UK), 2.5 μl of both forward and reverse primers (0.2 μM each), and 2.5 μl of nuclease‐free water (Qiagen). PCR conditions are as follows: 95°C for 15 min, 35 cycles of 95°C for 30 s, 54°C for 90 s, and 72°C for 90 s, and 72°C for 10 min, as instructed by the manufacturer (Qiagen). Each sample incorporated a unique combination of molecular identification (MID) tags (Binladen et al., 2007) that allowed for each skink to be identified after pooling and sequencing as per Brown et al. (2014). These 10‐bp fragments were added to both the forward and reverse primers for each sample, and thus, dietary taxon sequences could be assigned to individuals. PCR products were then run through a 2% agarose gel stained with SYBR^®^Safe (Thermo Fisher Scientific, Paisley, UK). Twelve negatives were included in each PCR run, 10 PCR negatives, and two extraction negatives. Additionally, two positive controls consisting of a standardized DNA concentration (4 ng/μl) of known invertebrate species likely absent from the study site (Supplementary Information S1) were used to control for tag‐jumping between samples in the filtering steps detailed below. PCR products were run in a Qiagen QIAxcel Advanced System (Qiagen) to measure relative DNA concentrations and later measured individually using a Qubit Fluorometer (Thermo Fisher Scientific) for more accurate determination of DNA concentrations. Each sample was then pooled based on the relative DNA concentrations of the amplicon of interest as measured by the QIAxcel Advanced System. Negative controls were pooled based on the average volume pooled for the skink samples. The pooling process involved adding a volume from each sample as a proportion of the sample with the highest concentration of DNA, to ensure approximate equimolarity of DNA from each sample. Each pool was cleaned using SPRIselect beads (Beckman Coulter, Brea, USA), with a left‐side size selection using a 1:1 ratio. After final elution, the pool was run on a Qubit Fluorometer, to measure DNA concentration (=49.6 ng/μl), as well as an Agilent 2200 TapeStation with D1000 ScreenTape (Agilent Technologies, Waldbronn) to check for significant levels of primer dimer, which were not found. This pool of MID‐tagged samples was then used for library preparation using the NEXTflex™ Rapid DNA‐Seq Kit following the manufacturer's instructions (Bioo Scientific Corp, Austin, TX, USA), which is suitable for pools with DNA concentrations of 1 ng–1 μg. A final DNA concentration was measured for the prepared library using a Qubit Fluorometer (=11.7 ng/μl) and was then sequenced on an Illumina MiSeq desktop sequencer (Illumina, San Diego, CA, USA) with a Nano cartridge using 2 × 250 bp paired reads (expected reads ≤1,000,000).

### 2.5 Bioinformatics

The Illumina Nano cartridge run generated 750,645 reads. High‐throughput sequencing data for the animal component of Telfair's skink diet followed the bioinformatic process of Drake et al. (2021): FastP (Chen et al., 2018) was used to check the quality of reads, discard poor quality reads (<Q30, <125 bp long or too many unqualified bases, denoted by “N”), trim reads to a minimum length of 300 bp and merge read pairs from Miseq files (R1 and R2). Read pairs were assigned to samples and demultiplexed using Mothur v1.39.5 (Schloss et al., 2009), after which MID‐tag and primer ends were removed. Unoise3 (Edgar, 2010) was used to remove replicates, denoise the sequences, and group identical sequences into zero‐radius operational taxonomic units (ZOTUs, which are clustered without % identity to avoid multiple species being nested within an OTU). Processed sequences were given taxonomic information from GenBank using BLASTn v2.7.1 (Camacho et al., 2009) with a 93% identity threshold. This threshold was chosen to capture the wide variety of invertebrates on Round Island to genus‐ or family‐level, most of which have not been barcoded or formally described. When more than one taxon was assigned to a sequence, we manually checked the feasibility for the presence of each taxon on Round Island by searching published articles, unpublished reports, and personal observations of species accounts. If these manual checks were inconclusive, we assigned the sequence to a higher taxonomic level (genus, family, order, etc.). MEGAN Community Edition v6.18.9 (Huson et al., 2016) was used to analyze the BLAST output and assign taxonomic identities to each ZOTU. Using the lowest e‐value (a value estimating the number of hits “expected” by chance when searching a database of a given size—in this instance anything <0.00001), the top hit was assigned to each sequence. Where top hits were taxonomic levels higher than species, these were manually checked and assigned to a feasible taxon or deleted from the analysis if erroneous. ZOTUs that were assigned to the same taxon were aggregated.

Data were cleaned for statistical analysis following the methods set out by Drake et al. (2021): The combined removal of the maximum read count in blanks and negative controls, and reads not meeting a predefined per sample threshold, removes both erroneous reads (laboratory contaminants and sequencing errors) that are likely to occur in low abundances mitigate tag‐jumping and bleeding of over‐represented taxa into other samples, while utilizing a per sample threshold and those arising through tag‐jumping and bleeding of over‐represented taxa into other samples removes erroneous reads (laboratory contaminants and sequencing errors) that are likely to occur in low abundances. The maximum read count of known contaminants and other obviously erroneous ZOTUs across the dataset was calculated as a percentage of their respective total sample read count, and any read counts less than this were removed. For this, a threshold of 0.3% was applied, removing low‐frequency laboratory contaminants and sequencing errors. Following this, the highest read count within a blank or negative per ZOTU was calculated and any ZOTU reads below this value were removed. In addition, we established an extra per‐ZOTU filtering step, which removed remaining erroneous taxa. The per‐ZOTU threshold was set to 0.74%. After these filters were applied, read counts were converted to presence–absence data for each sample. Nine samples were removed due to the absence of any dietary detections, leaving 73 samples to be taken forward for statistical analyses. Bioinformatic analysis for plant sequencing data followed Moorhouse‐Gann et al. (2021) (Supplementary Information S2).

After animal ZOTUs were given taxonomic information, status of each taxon relative to Round Island was determined for each by manually searching for relevant data in published articles, unpublished reports, and personal species accounts, and then classified as “cryptogenic,” “endemic,” “introduced,” or “native.” Cryptogenic species were defined as species that had no clear status, either because of poor taxonomic resolution, or because they may be known natives of the Indian Ocean islands, but their history on Round Island is unknown. Plant status was taken from Moorhouse‐Gann (2018; Moorhouse‐Gann et al., 2021).

### 2.6 Statistical analyses

Statistical analyses were conducted in R Statistical Software v4.1.0 (R Core Team, 2021) after data were converted to presence/absence within each sample. Basic characteristics of the diet were quantified by measuring frequency of occurrence. We aimed to reveal whether there were significant differences in the mean frequency of occurrence of dietary taxa from different taxonomic kingdoms (animals, plants) or status relative to Round Island (cryptogenic, endemic, introduced, native), hereafter “status.” Data were not normally distributed (Shapiro–Wilk test for normality: *W* = 0.64, *p* = <.001), and we therefore used two nonparametric Kruskal–Wallis tests, one each for kingdom and status, to determine whether there were significant differences in average consumption between categories of each variable.

We also wanted to quantify dietary diversity and show whether our samples could be used to sufficiently represent the broad dietary patterns of Telfair's skinks. Sample size and effort‐based standardization poorly represent the true diversity of communities because they fail to account for the species‐abundance distribution of the community being sampled (Cao et al., 2007; Roswell et al., 2021). We therefore used coverage‐based rarefaction and extrapolation rather than asymptotic species‐accumulation curves (Chao & Jost, 2012; Roswell et al., 2021) and robustly estimated species diversity using Hill diversity (Hill, 1973; Roswell et al., 2021). We define Hill diversity by the equation,

(1) $$D={\left(\sum_{i=1}^{S}p_{i}{\left(r_{i}\right)}^{\iota }\right)}^{\frac{1}{\iota }}$$

where *D* is diversity, *S* is number of species, *p_i_* is the proportion of all individuals that belong to species *i*, *r_i_* is the rarity of species *i*, defined as 1/*p_i_*, and *ι* is the exponent determining the rarity scale on which the mean is taken (Bullen, 2003; Hill, 1973; Roswell et al., 2021). Hill diversity is the generalized mean species rarity, and the exponent *ι* determines the sensitivity of the equation to rare species. *ι* of 1 uses the arithmetic mean rarity, or species richness (Hill‐richness), and is very sensitive to the rarest species; *ι* of 0 uses the geometric mean rarity, or the exponential of Shannon's entropy (Hill‐Shannon), and responds to both high and low rarity species; and *ι* of −1 uses the harmonic mean rarity, or the inverse of Simpson's index (Hill‐Simpson), and is most sensitive to the relative abundance of common species (Roswell et al., 2021). Coverage is a measure of how completely a community has been sampled and is an estimated proportion of the sampled individuals in the community that belong to species already detected (Chao & Jost, 2012). For example, a coverage of 0.85 denotes that 15% of the individuals in the community being sampled belong to species that have not been found. We computed these metrics in R package “iNEXT” (Hsieh et al., 2016).

Variation in diet composition was visualized with nonmetric multidimensional scaling (NMDS) in the “vegan” package (Oksanen et al., 2019) using the “metaMDS” function on a matrix of Jaccard distances, where we extracted three dimensions. Data were plotted using package “ggplot2” (Valero‐Mora, 2010). To illuminate whether sex, season, or their interaction affects Telfair's skink diet, R package “mvabund” was used (Wang et al., 2012). Multivariate generalized linear models (MGLMs) were run using the “manyglm” function with a Monte Carlo resampling method and “binomial” error family. The “step” function facilitated model selection where we selected the lowest AIC value denoting which model was most supported given the data.

## 3 RESULTS

There were 389 dietary presence counts belonging to 77 dietary taxa found across the 73 Telfair's skinks samples. Of these, 37 of 38 plant taxa were resolved to species due to extensive barcoding of the Round Island flora. The invertebrates of Round Island have not been described as extensively, and of the 39 dietary taxa detected, 20 were resolved to species, nine to genus, nine to family, and one to order. The invasive ant *P*. *megacephala* and a cryptogenic braconid wasp, *Heterospilus* sp., were the most frequently detected taxa, present in almost 40% of all Telfair's skink samples (Table 1; Table S2).

**TABLE 1:** Taxonomic information, frequency of occurrence *F*_O_ (%), and status relative to Round Island (cryptogenic, endemic, introduced, native) for all dietary taxa occurring in two or more Telfair's skink fecal samples

Kingdom	Phylum	Class	Order	Family	Dietary taxon	*F*_O_ (%)	Status
Animalia	Arthropoda	Arachnida	Araneae	Thomisidae	*Ozyptila claveata*	5.48	Introduced
		Crustacea	Isopoda	Porcellionidae	Porcellionidae sp.	34.25	Cryptogenic
		Insecta	Blattodea	Blaberidae	Blaberidae sp.	2.74	Cryptogenic
			Coleoptera	Coccinellidae	*Harmonia yedoensis*	20.55	Introduced
			Diptera	Drosophilidae	*Drosophila melanogaster*	2.74	Cryptogenic
					*Zaprionus indianus*	5.48	Introduced
				Tachinidae	*Chetogena* sp.	2.74	Cryptogenic
			Embioptera	Oligotomidae	*Oligotoma saundersii*	2.74	Introduced
			Hemiptera	Aleyrodidae	*Dialeurodes hongkongensis*	2.74	Introduced
				Rhyparochromidae	Rhyparochromidae	8.22	Cryptogenic
			Hymenoptera	Apidae	*Inquilina* sp.	2.74	Native
				Braconidae	*Heterospilus* sp.	39.73	Cryptogenic
				Formicidae	*Brachymyrmex cordemoyi*	19.18	Introduced
					Formicidae sp.	2.74	Cryptogenic
					*Monomorium floricola*	2.74	Introduced
					*Pheidole megacephala*	39.73	Introduced
				Platygastridae	Platygastridae sp.	2.74	Cryptogenic
Plantae	Angiosperms	Eudicots	Asterales	Asteraceae	*Erigeron bonariensis*	5.48	Introduced
					*Tridax procumbens*	10.96	Introduced
				Goodeniaceae	*Scaevola taccada*	10.96	Native
			Caryophyllales	Amaranthaceae	*Achyranthes aspera*	19.18	Introduced
					*Amaranthus viridis*	2.74	Introduced
				Nyctaginaceae	*Boerhavia* sp.	19.18	Native
			Celastrales	Celastraceae	*Gymnosporia pyria*	2.74	Endemic
			Fabales	Fabaceae	*Desmodium incanum*	6.85	Introduced
					*Gagnebina pterocarpa*	10.96	Native
			Gentianales	Apocynaceae	*Vincetoxicum confusum*	12.33	Native
			Lamiales	Lamiaceae	*Premna serratifolia*	5.48	Native
			Malpighiales	Euphorbiaceae	*Euphorbia thymifolia*	4.11	Cryptogenic
				Passifloraceae	*Passiflora suberosa*	15.07	Introduced
				Phyllanthaceae	*Margaritaria anomala*	4.11	Endemic
			Malvales	Malvaceae	*Abutilon indicum*	35.62	Introduced
					*Hibiscus tiliaceus*	5.48	Native
					*Hilsenbergia petiolaris*	4.11	Native
			Myrtales	Myrtaceae	*Eugenia lucida*	4.11	Endemic
			Solanales	Convolvulaceae	*Ipomoea pes‐caprae*	21.92	Native
				Solanaceae	*Solanum lycopersicum*	4.11	Introduced
					*Solanum nigrum*	17.81	Introduced
		Monocots	Arecales	Arecaceae	*Latania loddigesii*	32.88	Endemic
			Poales	Poaceae	*Cenchrus echinatus*	13.70	Introduced
					*Chloris barbata*	2.74	Introduced
					*Dactyloctenium ctenoides*	4.11	Native
					*Digitaria horizontalis*	12.33	Introduced

Note

Cryptogenic taxa had no clear status, either because of poor taxonomic resolution, or because they may be known natives of the Indian Ocean islands, but their history on Round Island is unknown.

**FIGURE 4:**
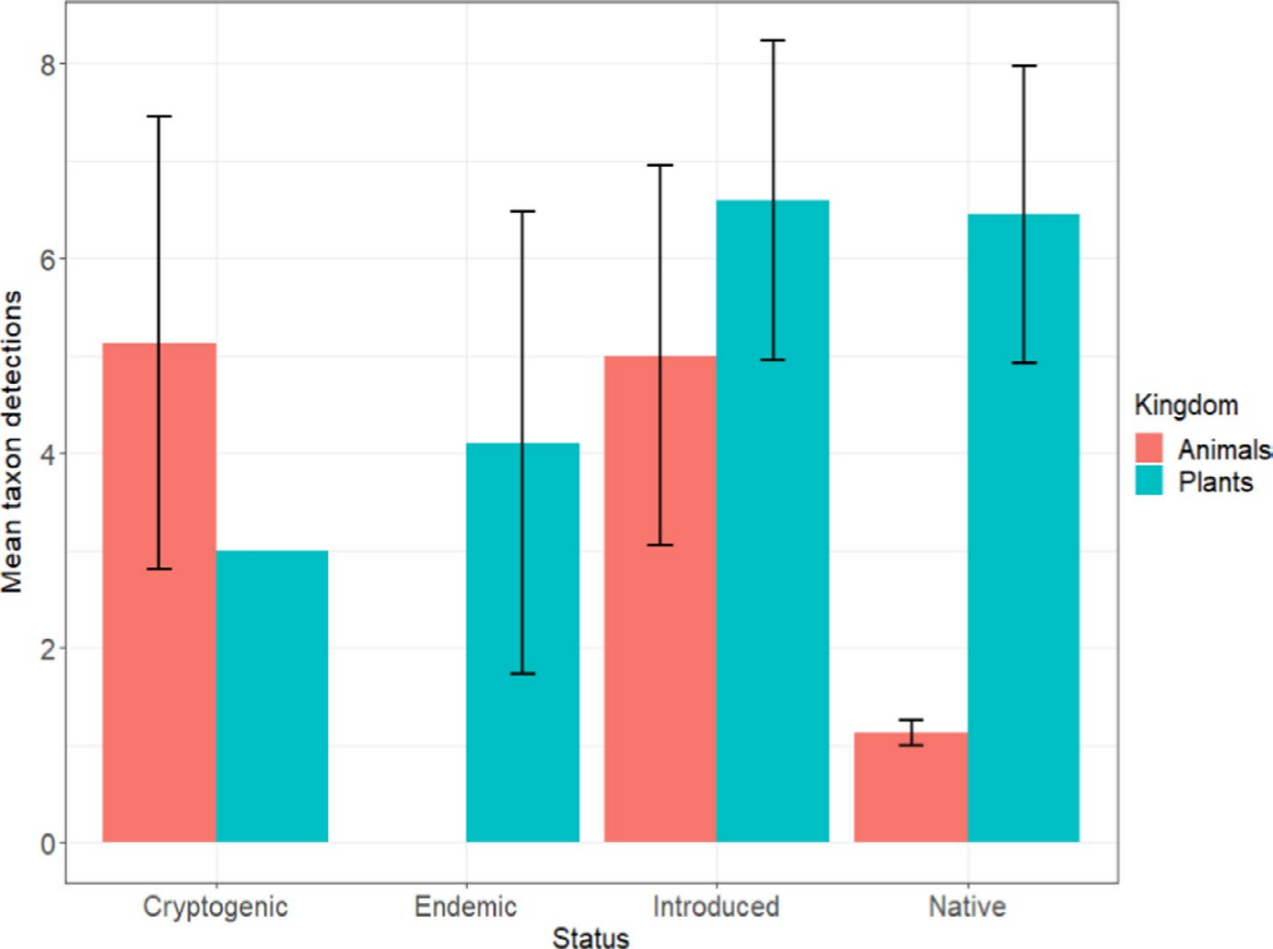
Mean (± *SE*) dietary taxon detections of Telfair's skinks (*Leiolopisma telfairii*) by dietary taxon status relative to Round Island (cryptogenic, endemic, introduced, or native) and taxonomic kingdom. Means within each category were calculated by dividing total detections by the number of dietary taxa detected. Note: There were no endemic animal taxa recorded in the diet of the skinks and only one cryptogenic plant was detected (no SE bar)

**FIGURE 5:**
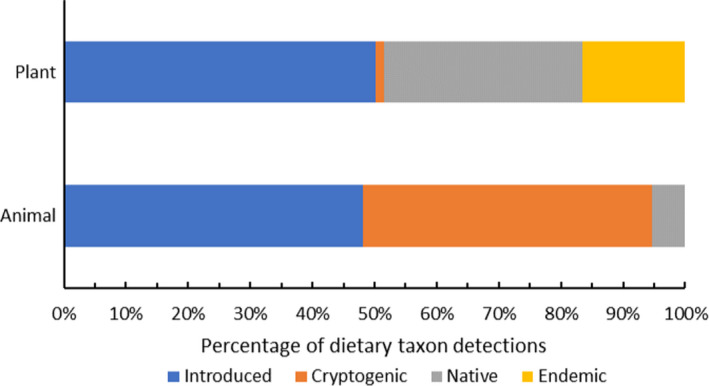
Plant and animal dietary taxon detections as a percentage of total detections by status relative to Round Island (cryptogenic, endemic, introduced, native)

Our Kruskal–Wallis tests showed that mean number of detections per dietary taxon was not significantly affected by status relative to Round Island (*χ^2^* (3) = 1.51, *p* = .68), but taxonomic kingdom did show a significant effect (*χ^2^* (1) = 6.33, *p* = .012), where plants were consumed more frequently on average per taxon than animals (mean consumption per dietary taxon (± SE): animals = 4.26 (± 1.2), plants = 5.87 (± 1.03); Figure 4, Table S3). Introduced taxa were cumulatively consumed more frequently than all other status categories, accounting for 49.4% of all detections, while cryptogenic and native taxa accounted for 20.6% each, and endemic taxa 9.5% (Figure 5).

**FIGURE 6:**
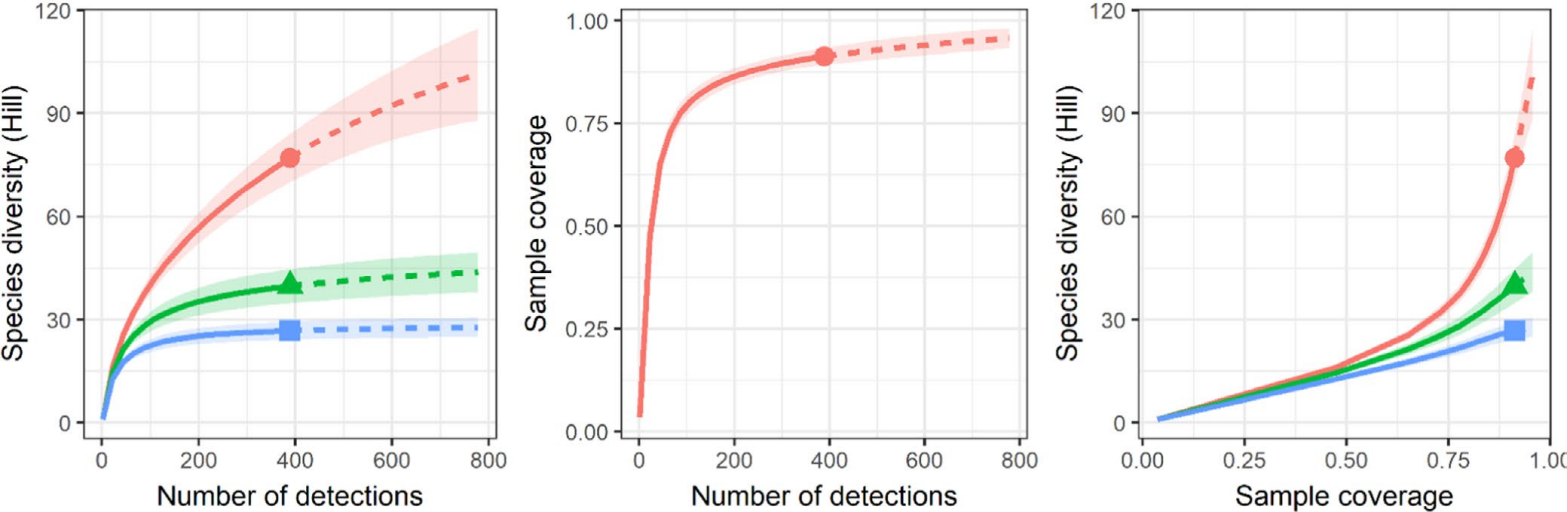
Dietary species diversity found in Telfair's skink fecal samples and the level of community coverage provided. Line colors denote values of the exponent *ι* that determines the rarity scale of different diversity estimates: Hill‐richness, *ι* = 1, red line with terminal circle; Hill‐Shannon, *ι* = 0, green line with terminal triangle; Hill‐Simpson, *ι* = −1, blue line with terminal square. Solid lines = observed, dashed lines = extrapolated. Confidence intervals (95%) are denoted by shading around the line. Left: species diversity by number of dietary detections. Centre: sample coverage by number of dietary detections. Right: species diversity by sample coverage

We computed dietary diversity (Figure 6) and found that Hill‐richness (*ι* = 1) provided the highest diversity estimate in contrast to both Hill‐Shannon (*ι* = 0) and Hill‐Simpson (*ι* = −1) (Figure 6, left plot). Together, these diversity estimates suggest Telfair's skinks consume many rarely eaten individual species instead of evenly consuming dietary taxa or just a few commonly eaten species. We estimated that our sampling provided 95.7% (±95% CI: 2.6%) coverage of the dietary community (Figure 6, center and right plots), meaning that 4.3% of individuals in the theoretical diet belonged to species we did not detect.

**FIGURE 7:**
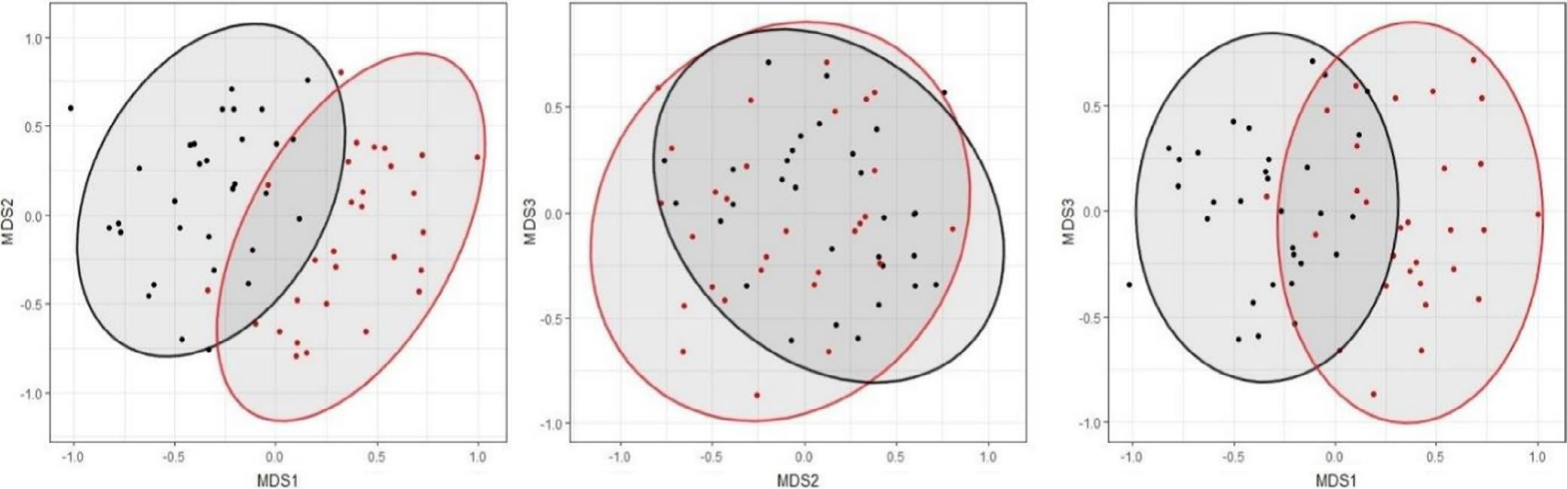
Pairwise biplots from non‐metric multidimensional scaling (NMDS) analysis in three dimensions (stress = 0.161). Point and ellipse colors denote seasons: black = dry; red = wet. Ellipses show 95% of data rotated to the direction of maximum spread

MGLMs showed that diet composition differed significantly between season (Wald = 259.88, *p* = <.001), sex (Wald = 226.22, *p* = <.001), and their interaction (Wald = 30.54, *p* = .027). Diet composition between seasons was visualized using NMDS (Figure 7) (stress = 0.161). Three species showed at least one significant GLM result: *Abutilon indicum* (season*sex: Wald = 9.035, *p* = .031), *A*. *aspera* (season: Wald = 31.097, *p* = <.001), and *L*. *loddigesii* (sex: Wald = 25.161, *p* = .002; season: Wald = 31.213, *p* = <.001).

## 4 DISCUSSION

### 4.1 Key trophic interactions and dietary diversity

These findings corroborate previous analyses of diet, suggesting Telfair's skinks are generalist omnivores that consume a wide range of animal and plant taxa (Brown et al., 2014; Cole, Goder, et al., 2018; Moorhouse‐Gann et al., 2021; Pernetta et al., 2005). Moreover, our diversity estimates suggest Telfair's skinks consume many species infrequently instead of consuming taxa evenly. This study achieved a greater taxonomic resolution compared to previous molecular analyses of Telfair's skink diet (Brown et al., 2014; Pernetta et al., 2005), resolving almost all plant taxa and nearly half of the invertebrate taxa to species‐level. In contrast, previous analyses could not resolve dietary invertebrate taxa to species level at all.

Dietary taxa consumed once or twice form a large component of Telfair's skink diet, suggesting that they may opportunistically consume many rare species, but rely on a few other species for more consistent nutrition, which may also be seasonal. For example, *L*. *loddigesii* was the most frequently consumed native dietary taxon, being present in 32.9% of samples, and all detections occurred in the dry season. These trees form the dominant native habitat type on Round Island and produce fruits, pollen, and nectar that skinks are known to readily consume (Cole, Goder, et al., 2018; Cole, Mootoocurpen, et al., 2018). Since all detections of *L*. *loddigesii* occurred in the dry season, when fruits are produced, this suggests they are a seasonal nutritional resource for Telfair's skinks. Moreover, female skinks accounted for 60% of all *L*. *loddigesii* detections and fruits may therefore be disproportionately important or attractive to female skinks. Telfair's skinks typically mate throughout the dry season (Cole, Goder, et al., 2018) and *L*. *loddigesii* consumption may provide essential nutrition or minerals for growth and/or egg production in females. In contrast, 34 dietary taxa were detected only once, 12 taxa were detected twice, and six taxa were detected three times, cumulatively representing almost a fifth of total detections. Our diversity estimates suggest Telfair's skinks consume many dietary taxa at relatively low frequencies. This may represent the foraging behavior of Telfair's skinks whereby they feed on a few species regularly but supplement their diet by opportunistically consuming a much greater diversity of other animals and plants, albeit at a lower frequency for each taxon.

There are an estimated 46,000 Telfair's skinks on Round Island (Cole, Mootoocurpen, et al., 2018), with an estimated 210 skinks per ha island‐wide. This represents a major component of total animal biomass. Given the abundance and size of Telfair's skinks, these results show they are likely to represent a major top‐down pressure on the ecological network through their dietary generalism.

### 4.2 Prevalence of introduced taxa

Overall, introduced taxa formed the primary component of Telfair's skink diet as measured by frequency of occurrence. The majority of dietary detections and richness were of introduced taxa, accounting for almost half in both cases. Therefore, this study illuminates that introduced taxa have become a large part of the diet of a globally threatened endemic species. However, for some taxa it is unclear whether skinks rely on them for nutrition, and this is a broader issue in dietary metabarcoding studies because sequencing data cannot convey nutritional information (Alberdi et al., 2019; Lamb et al., 2019). For example, the introduced ant *P*. *megacephala* is present in 39.7% of samples, but may be a distasteful meal for Telfair's skinks. On Round Island, *P*. *megacephala* is hyperabundant and found in every habitat type in this study. Predation may not provide a cost‐effective nutritional reward to an unspecialized ant‐eating vertebrate given that the ant is very small compared to Telfair's skinks and ants typically possess unpleasant and/or harmful compounds (Schmidt, 2009). If these ants truly are deleterious to skinks, their high frequency of occurrence in the diet could be explained by accidental consumption. Accidental consumption may occur when skinks consume food items that have been colonized by ants, which typically occurs rapidly on Round Island. Another explanation is through secondary predation, which entails detection of food items in the digestive system of primary skink prey. Both accidental consumption and secondary predation may complicate interpretation of dietary analyses using HTS (Robeson et al., 2018; Silva et al., 2019; Tercel et al., 2021). Nevertheless, even accidental ingestion of some species could provide nutritional benefits to skinks.

With roughly half of all dietary detections originating from introduced species, non‐native taxa appear to be a dominant part of Telfair's skink diet. It may be that the original components of the diet have been lost after Round Island suffered severe habitat destruction and have been subsequently replaced by non‐native species. Equally, the availability or nutritional value of non‐native species may be relatively higher than existing native food.

Cryptogenic invertebrates represented almost 20% of all dietary detections and therefore likely represent an important component of Telfair's skink diet. In reality, cryptogenic species are either introduced or native, but this information is lost without adequate taxonomic information. Unfortunately, many of the invertebrates on Round Island remain undescribed and are absent from barcode reference libraries, which presents a problem when assigning an origin to ZOTUs that do not resolve to species‐level. Furthermore, many undescribed invertebrate species categorized as cryptogenic may be endemic and globally threatened. The use of a 93% identity threshold in this study permits assignment of sequences to a higher taxonomic level for species absent from barcode libraries, that is, to genus or family, but this does not solve how to assign a dietary taxon a status. Our study deliberately took a conservative approach to assigning a status category to taxa, but it may be more likely for cryptogenic species to be native than introduced. This is because many introduced species are globally common and have been barcoded, whereas endemic species have not. Work to formally describe, identify, and barcode Round Island invertebrate species is therefore essential to disentangling this problem and to more fully describing the ecology of Round Island.

### 4.3 Seasonal and sex differences

The presence of plant species in the diet of the skinks solely or primarily in one season, such as *L*. *loddigesii* (all 24 detections in the dry season) and *A*. *aspera* (all 14 detections in the wet season), partly explains the strong difference in diet composition between seasons. Broad seasonal differences in diet are further explained by animal taxa being a much greater component of skink diet in the dry season, where 61.4% of animal prey detections occurred. Despite this, most animal taxa were consumed across both seasons, while most plant taxa were consumed primarily in only one season. This confirms that Telfair's skinks rely on different dietary taxa at different times of the year at a broad scale, with only modest overlap in composition (Figure 7). Seasonal differences in diet composition very likely arise because of changes in the availability of food sources between the markedly different seasons in Mauritius (Senapathi et al., 2010).

Two dietary taxa were consumed at different rates between male and female skinks: *L*. *loddigesii*, as discussed above, and *A*. *indicum*, which was the most frequently consumed plant, present in 35.6% of samples. *Abutilon indicum* is native to tropical and subtropical Asia but has been widely transported across the global tropics and is locally abundant over much of Round Island. It produces flowers and seeds that may be attractive to skinks year‐round. Male skinks consumed *A*. *indicum* more frequently in the wet season (71.4% of detections) than the dry season (28.6%), but the inverse was true of female skinks (100% of detections in the dry season). A possible explanation is that plant tissues of *A*. *indicum* (e.g., flower, nectar, seeds) are consumed differently between sexes. Because the availability of these varies throughout the year, it may mean that males and females consume *A*. *indicum* differently between seasons. Exactly how the tissue types of *A*. *indicum* may differentially benefit male and female skinks requires further study.

Understanding the nutritional requirements between sexes could be an important factor governing the success of skink translocations. Although we broadly see that male and female skinks consume the same species, we show that female skinks might rely more on certain species during the breeding season, which is a pivotal period in any reintroduction program.

### 4.4 Limitations

The general limitations of dietary metabarcoding have been reviewed extensively by other authors (Alberdi et al., 2019; Lamb et al., 2019; Nielsen et al., 2018; Taberlet et al., 2018), but we also identified some study‐specific limitations. This study converts sequence data to presence/absence and subsequently frequency of occurrence. We believe this is the most robust interpretation of sequencing data, because sequencing output only very weakly correlates with biomass in a sample (Deagle et al., 2019; Lamb et al., 2019). Nevertheless, frequency of occurrence therefore also omits how much biomass is consumed in each sample and, thus, a dietary taxon may appear frequently between samples but not contribute proportionately to the nutrition of the consumer.

As discussed above, the very high prevalence of introduced ants in Telfair's skink diet is difficult to explain ecologically with any certainty. These have not been observed to be directly eaten by the skinks, but are ubiquitous over Round Island, and colonize food resources rapidly. Moreover, a very frequently found tiny (<2 mm) cryptogenic braconid wasp, *Heterospilus* sp., seems unlikely to be actively preyed upon by adult Telfair's skinks. Accidental consumption or secondary predation might explain these detections, as has been seen in other dietary metabarcoding studies (Silva et al., 2019), and have been identified as a potential source of error that may disproportionately complicate the interpretation of dietary analyses of omnivores (Tercel et al., 2021). With an aim to tease apart some of these issues, we conducted a co‐occurrence analysis (Supplementary Information 3; Figure S1) but found no clear ecological patterns that explain these detections. Indeed, co‐occurrence analyses may be used as an exploratory element in ecological studies but cannot provide strong evidence to support ecological hypotheses in this context (Blanchet et al., 2020), and may not facilitate interpretation (Tercel et al., 2021).

Omnivores can exert top‐down effects across the breadth and depth of ecological networks, and studying their diet is therefore valuable to the field of ecology. However, omnivorous diets require extra caution when inferring ecological conclusions from sequencing results given that some detections may not be ecologically meaningful. In this study, it may be that Telfair's skinks *are* directly consuming both *P*. *megacephala* and *Heterospilus* sp., but it remains unclear whether this is true from conflicting behavioral observations of Telfair's skinks and our inconclusive co‐occurrence analysis that does not provide alternative ecological explanations.

### 4.5 Concluding remarks

Our study represents one of only a few complete dietary analyses of omnivores using DNA metabarcoding (but see De Barba et al., 2014; Ducotterd et al., 2021; Robeson et al., 2018; Silva et al., 2019) and the first study examining the omnivorous diet of a threatened endemic reptile. We found that Telfair's skinks consume a few species regularly and many species rarely. We also found that Telfair's skinks rely on *L*. *loddigesii* fruits during the dry season on Round Island, coinciding with when breeding takes place. Though restored habitat on Round Island does not cover the whole island, extensive habitat regeneration efforts since 2002 have led to the continued recovery of the forests on Round Island, and this bodes well for the future of the skinks. Nevertheless, almost half of all dietary detections were of introduced species and it is increasingly clear that the ecological impacts of introduced species on Round Island are multifaceted, with some species acting as *de facto* ecological replacements. Telfair's skinks have probably incorporated introduced species into their diet as a replacement for the many species that were presumably lost by severe habitat destruction on Round Island. Further studies showing the mechanisms by which Telfair's skinks consume certain species would help to provide more confident ecological explanations for some of these interactions.

Although many introduced species damage native ecosystems, some species may benefit regenerating habitats and their inhabitants, particularly in the context of island restoration. This study shows that many introduced species of animal and plant contribute positively to providing nutritional subsidies to a globally threatened endemic omnivore. Positive effects of introduced species must therefore be weighed up against potential negative consequences of colonization for the ecosystem. This is pertinent for conservation managers to consider when restoring native habitats and controlling introduced species, especially when threatened animal species may be consuming introduced taxa in the absence of lost native food resources.

## CONFLICT OF INTEREST

The authors declare there are no conflicts of interest.

## AUTHOR CONTRIBUTION

**Maximillian P. T. G. Tercel:** Data curation (equal); Formal analysis (lead); Investigation (lead); Methodology (equal); Project administration (lead); Resources (equal); Software (equal); Validation (equal); Visualization (equal); Writing – original draft (lead); Writing – review & editing (lead). **Rosemary J. Moorhouse‐Gann:** Data curation (equal); Funding acquisition (lead); Investigation (equal); Methodology (equal); Project administration (supporting); Writing – review & editing (supporting). **Jordan P. Cuff:** Formal analysis (equal); Investigation (equal); Methodology (equal); Writing – review & editing (supporting). **Lorna E. Drake:** Formal analysis (equal); Investigation (equal); Methodology (equal); Writing – review & editing (supporting). **Nik C. Cole:** Conceptualization (equal); Funding acquisition (equal); Investigation (equal); Methodology (equal); Project administration (equal); Resources (equal); Supervision (equal); Writing – review & editing (equal). **Martine Goder:** Investigation (equal); Methodology (supporting); Project administration (equal). **Rouben Mootoocurpen:** Investigation (equal); Methodology (equal); Project administration (supporting). **William O. C. Symondson:** Conceptualization (lead); Funding acquisition (lead); Methodology (equal); Project administration (lead); Supervision (lead); Writing – review & editing (equal).

## Supporting information

Supplementary Material

## Data Availability

Animal sequencing data are available from Zenodo: https://zenodo.org/record/5476197#.YbHafNDP02y. Plant sequencing data are deposited in the SCBI Sequence Read Archive.

## References

[ece38484-bib-0001] Alberdi, A. , Aizpurua, O. , Bohmann, K. , Gopalakrishnan, S. , Lynggaard, C. , Nielsen, M. , & Gilbert, M. T. P. (2019). Promises and pitfalls of using high‐throughput sequencing for diet analysis. Molecular Ecology Resources, 19(2), 327–348. 10.1111/1755-0998.12960 30358108

[ece38484-bib-0002] Ando, H. , Setsuko, S. , Horikoshi, K. , Suzuki, H. , Umehara, S. , Inoue‐Murayama, M. , & Isagi, Y. (2013). Diet analysis by next‐generation sequencing indicates the frequent consumption of introduced plants by the critically endangered red‐headed wood pigeon (*Columba janthina nitens*) in oceanic island habitats. Ecology and Evolution, 3(12), 4057–4069. 10.1002/ece3.773 24324859 PMC3853553

[ece38484-bib-0003] Arbetman, M. P. , Meeus, I. , Morales, C. L. , Aizen, M. A. , & Smagghe, G. (2013). Alien parasite hitchhikes to Patagonia on invasive bumblebee. Biological Invasions, 15(3), 489–494. 10.1007/s10530-012-0311-0

[ece38484-bib-0004] Baldock, K. C. R. , Goddard, M. A. , Hicks, D. M. , Kunin, W. E. , Mitschunas, N. , Osgathorpe, L. M. , Potts, S. G. , Robertson, K. M. , Scott, A. V. , Stone, G. N. , Vaughan, I. P. , & Memmott, J. (2015). Where is the UK's pollinator biodiversity? The importance of urban areas for flower‐visiting insects. Proceedings of the Royal Society B: Biological Sciences, 282(1803), 20142849. 10.1098/rspb.2014.2849 PMC4345454 25673686

[ece38484-bib-0005] Binladen, J. , Gilbert, M. T. P. , Bollback, J. P. , Panitz, F. , Bendixen, C. , Nielsen, R. , & Willerslev, E. (2007). The use of coded PCR primers enables high‐throughput sequencing of multiple homolog amplification products by 454 parallel sequencing. PLoS One, 2(2), 1–9. 10.1371/journal.pone.0000197 PMC1797623 17299583

[ece38484-bib-0006] Blanchet, F. G. , Cazelles, K. , & Gravel, D. (2020). Co‐occurrence is not evidence of ecological interactions. Ecology Letters, 23(7), 1050–1063. 10.1111/ele.13525 32429003

[ece38484-bib-0007] Bonin, M. , Dussault, C. , Taillon, J. , Lecomte, N. , & Côté, S. D. (2020). Combining stable isotopes, morphological, and molecular analyses to reconstruct the diet of free‐ranging consumers. Ecology and Evolution, 10(13), 6664–6676. 10.1002/ece3.6397 32724540 PMC7381590

[ece38484-bib-0008] Brown, D. S. , Burger, R. , Cole, N. , Vencatasamy, D. , Clare, E. L. , Montazam, A. , & Symondson, W. O. C. (2014). Dietary competition between the alien Asian Musk Shrew (*Suncus murinus*) and a re‐introduced population of Telfair’s Skink (*Leiolopisma telfairii*). Molecular Ecology, 23(15), 3695–3705. 10.1111/mec.12445 24033506

[ece38484-bib-0009] Bullen, P. S. (2003). Handbook of means and their inequalities. Kluwer Academic Publishers.

[ece38484-bib-0010] Camacho, C. , Coulouris, G. , Avagyan, V. , Ma, N. , Papadopoulos, J. , Bealer, K. , & Madden, T. L. (2009). BLAST+: Architecture and applications. BMC Bioinformatics, 10, 1–9. 10.1186/1471-2105-10-421 20003500 PMC2803857

[ece38484-bib-0011] Cao, Y. , Hawkins, C. P. , Larsen, D. P. , & Van Sickle, J. (2007). Effects of sample standardization on mean species detectabilities and estimates of relative differences in species richness among assemblages. The American Naturalist, 170(3), 381–395. 10.1086/520117 17879189

[ece38484-bib-0012] Chao, A. , & Jost, L. (2012). Coverage‐based rarefaction and extrapolation: Standardizing samples by completeness rather than size. Ecology, 93(12), 2533–2547. 10.1890/11-1952.1 23431585

[ece38484-bib-0013] Cheke, A. , & Hume, J. P. (2008). Lost land of the dodo: An ecological history of mauritius, réunion & rodrigues, 1st ed. Bloomsbury.

[ece38484-bib-0014] Chen, S. , Zhou, Y. , Chen, Y. , & Gu, J. (2018). Fastp: An ultra‐fast all‐in‐one FASTQ preprocessor. Bioinformatics, 34(17), i884–i890. 10.1093/bioinformatics/bty560 30423086 PMC6129281

[ece38484-bib-0015] Clavero, M. , & Garcia‐Berthou, E. (2005). Invasive species are a leading cause of animal extinctions. Trends in Ecology and Evolution 20(3):110. *TRENDS in Ecology and Evolution* **19**(January):17071. 16701353 10.1016/j.tree.2005.01.003

[ece38484-bib-0016] Cole, N. , Goder, M. , Premanand, R. , Bachraz, V. , & Mootoocurpen, R. (2018). Leiolopisma telfairii . The IUCN Red List of Threatened Species, 2018, 8235.

[ece38484-bib-0017] Cole, N. C. , & Harris, S. (2011). Environmentally‐induced shifts in behavior intensify indirect competition by an invasive gecko in Mauritius. Biological Invasions, 13(9), 2063–2075. 10.1007/s10530-011-0025-8

[ece38484-bib-0018] Cole, N. , Mootoocurpen, R. , & Nundlaul, V. (2018). Relative density estimates of Round Island’s reptiles. Journal of the Royal Society of Arts and Sciences of Mauritius 1(Vinson 1949):1–113.

[ece38484-bib-0019] Cuff, J. P. , Drake, L. E. , Tercel, M. P. T. G. , Stockdale, J. E. , Orozco‐terWengel, P. , Bell, J. R. , Vaughan, I. P. , Müller, C. T. , & Symondson, W. O. C. (2021). Money spider dietary choice in pre‐ and post‐harvest cereal crops using metabarcoding. Ecological Entomology, 46(2), 249–261. 10.1111/een.12957

[ece38484-bib-0020] De Barba, M. , Miquel, C. , Boyer, F. , Mercier, C. , Rioux, D. , Coissac, E. , & Taberlet, P. (2014). DNA metabarcoding multiplexing and validation of data accuracy for diet assessment: Application to omnivorous diet. Molecular Ecology Resources, 14(2), 306–323. 10.1111/1755-0998.12188 24128180

[ece38484-bib-0021] Deagle, B. E. , Thomas, A. C. , McInnes, J. C. , Clarke, L. J. , Vesterinen, E. J. , Clare, E. L. , Kartzinel, T. R. , & Eveson, J. P. (2019). Counting with DNA in metabarcoding studies: How should we convert sequence reads to dietary data? Molecular Ecology, 28(2), 391–406. 10.1111/mec.14734 29858539 PMC6905394

[ece38484-bib-0022] Drake, L. E. , Cuff, J. P. , Young, R. E. , Marchbank, A. , Chadwick, E. A. , & Symondson, W. O. C. (2021). An assessment of minimum sequence copy thresholds for identifying and reducing the prevalence of artefacts in dietary metabarcoding data. Methods in Ecology and Evolution, 1–17. 10.1111/2041-210X.13780

[ece38484-bib-0023] Ducotterd, C. , Crovadore, J. , Lefort, F. , Rubin, J. , & Ursenbacher, S. (2021 ). A powerful long metabarcoding method for the determination of complex diets from faecal analysis of the European pond turtle (*Emys orbicularis*, L. 1758). Molecular Ecology Resources, 21(2):433–447. 10.1111/1755-0998.13277 33047508 PMC7821331

[ece38484-bib-0024] Edgar, R. C. (2010). Search and clustering orders of magnitude faster than BLAST. Bioinformatics, 26(19), 2460–2461. 10.1093/bioinformatics/btq461 20709691

[ece38484-bib-0025] Elbrecht, V. , & Leese, F. (2017). Development and validation of DNA metabarcoding COI primers for aquatic invertebrates using the R package ‘PrimerMiner’. Methods in Ecology and Evolution, 8(5), 622–626. 10.7287/peerj.preprints.2044v1

[ece38484-bib-0026] Griffiths, C. J. , Jones, C. G. , Hansen, D. M. , Puttoo, M. , Tatayah, R. V. , Müller, C. B. , & Harris, S. (2010). The use of extant non‐indigenous tortoises as a restoration tool to replace extinct ecosystem engineers. Restoration Ecology, 18(1), 1–7. 10.1111/j.1526-100X.2009.00612.x

[ece38484-bib-0027] Hill, M. O. (1973). Diversity and evenness: A unifying notation and its consequences. Ecology, 54(2), 427–432. 10.2307/1934352

[ece38484-bib-0028] Hsieh, T. C. , Ma, K. H. , & Chao, A. (2016). iNEXT: an R package for rarefaction and extrapolation of species diversity (h ill numbers) McInerny, G. (ed.). Methods in Ecology and Evolution, 7(12):1451–1456. 10.1111/2041-210X.12613

[ece38484-bib-0029] Huson, D. H. , Beier, S. , Flade, I. , Górska, A. , El‐Hadidi, M. , Mitra, S. , & Tappu, R. (2016). MEGAN community edition ‐ interactive exploration and analysis of large‐scale microbiome sequencing data. PLOS Computational Biology, 12(6), e1004957. 10.1371/journal.pcbi.1004957 27327495 PMC4915700

[ece38484-bib-0030] IUCN . (2020). The IUCN Red List of Threatened Species. Version 2020‐2. https://www.iucnredlist.org Downloaded on 09 July

[ece38484-bib-0031] Jones, C. G. (1993). The ecology and conservation of Mauritian skinks. Proceedings of the Royal Society of Arts and Sciences of Mauritius, 5(3), 71–95.

[ece38484-bib-0032] Jones, C. G. (2008). Practical conservation on mauritius and rodrigues: steps towards the restoration of devastated ecosystems. In A. Cheke & J. P. Hume (Eds.), Lost land of the dodo: An ecological history of mauritius, Réunion & Rodrigues. T. & A. D. Poyser.

[ece38484-bib-0033] Kaiser‐Bunbury, C. N. , Valentin, T. , Mougal, J. , Matatiken, D. , & Ghazoul, J. (2011). The tolerance of island plant‐pollinator networks to alien plants. Journal of Ecology, 99(1), 202–213. 10.1111/j.1365-2745.2010.01732.x

[ece38484-bib-0034] Kato, Y. , & Suzuki, T. (2005). Introduced animals in the diet of the Ogasawara buzzard, an endemic insular raptor in the Pacific Ocean. Journal of Raptor Research, 39, 173–179.

[ece38484-bib-0035] Lamb, P. D. , Hunter, E. , Pinnegar, J. K. , Creer, S. , Davies, R. G. , & Taylor, M. I. (2019). How quantitative is metabarcoding : A meta ‐ analytical approach. Molecular Ecology, 28:420–430. 10.1111/mec.14920 30408260 PMC7379500

[ece38484-bib-0036] Lewis, J. G. E. , Daszak, P. , Jones, C. G. , Cottingham, J. D. , Wenman, E. , & Maljkovic, A. (2010). Field observations on three scolopendrid centipedes from Mauritius and Rodrigues (Indian Ocean) (Chilopoda: Scolopendromorpha). International Journal of Myriapodology, 3(1), 123–137. 10.1163/187525410x12578602960425

[ece38484-bib-0037] Li, Y. , Ke, Z. , Wang, S. , Smith, G. R. , & Liu, X. (2011). An exotic species is the favorite prey of a native enemy. PLoS One, 6(9), 10.1371/journal.pone.0024299 PMC3167836 21915306

[ece38484-bib-0038] Luque, G. M. , Bellard, C. , Bertelsmeier, C. , Bonnaud, E. , Genovesi, P. , Simberloff, D. , & Courchamp, F. (2014). The 100th of the world’s worst invasive alien species. Biological Invasions, 16(5), 981–985. 10.1007/s10530-013-0561-5

[ece38484-bib-0039] Memmott, J. , Fowler, S. V. , Paynter, Q. , Sheppard, A. W. , & Syrett, P. (2000). The invertebrate fauna on broom, *Cytisus scoparius*, in two, native and two exotic habitats. Acta Oecologica, 21(3), 213–222. 10.1016/S1146-609X(00)00124-7

[ece38484-bib-0040] Merton, D. (1987). Eradication of rabbits from Round Island, Mauritius: A conservation success story. Dodo, 24, 19–43.

[ece38484-bib-0041] Moldowan, P. D. , Copsey, J. A. , Zuël, N. , Tatayah, V. , & Cole, N. (2016). Sticks and stones: notes on the ecology and conservation of an endemic stick insect (*Apterograeffea marshallae*) and the restoration of an island ecosystem. Phelsuma, 24, 72–79.

[ece38484-bib-0042] Moorhouse‐Gann, R. J. (2018). Ecological replacement as a restoration tool: Disentangling the impacts and interactions of aldabra giant tortoises (*Aldabrachelys gigantea*) using DNA metabarcoding a thesis submitted to Cardiff University for the Degree of Doctor By. Cardiff University.

[ece38484-bib-0043] Moorhouse‐Gann, R. J. , Dunn, J. C. , de Vere, N. , Goder, M. , Cole, N. , Hipperson, H. , & Symondson, W. O. C. (2018). New universal ITS2 primers for high‐resolution herbivory analyses using DNA metabarcoding in both tropical and temperate zones. Scientific Reports, 8(1), 8542. 10.1038/s41598-018-26648-2 29867115 PMC5986805

[ece38484-bib-0044] Moorhouse‐Gann, R. , Vaughan, I. P. , Cole, N. , Goder, M. , Tatayah, V. , Jones, C. , Mike, D. , Young, R. P. , Bruford, M. W. , Rivers, M. C. , Hipperson, H. , Russo, I.‐R. M. , Stanton, D. W. G. , & Symondson, W. O. C. (2021). Impacts of ecological replacement on an island ecosystem. Journal of Applied Ecology. 10.1111/1365-2664.14096

[ece38484-bib-0045] Nielsen, J. M. , Clare, E. L. , Hayden, B. , Brett, M. T. , & Kratina, P. (2018). Diet tracing in ecology: Method comparison and selection Gilbert, M. T. P. (ed.). Methods in Ecology and Evolution, 9(2), 278–291. 10.1111/2041-210X.12869

[ece38484-bib-0046] North, S. G. , Bullock, D. J. , & Dulloo, M. E. (1994). Changes in the vegetation and reptile populations on Round Island, Mauritius, following eradication of rabbits. Biological Conservation, 67(1), 21–28. 10.1016/0006-3207(94)90004-3

[ece38484-bib-0047] O’Dowd, D. J. , Green, P. T. , & Lake, P. S. P. (2003). Invasional ‘meltdown’ on an oceanic island. Ecology Letters, 6(9), 812–817. 10.1046/j.1461-0248.2003.00512.x

[ece38484-bib-0048] Oksanen, J. , Blanchet, F. G. , Friendly, M. , Kindt, R. , Legendre, P. , McGlinn, D. , Minchin, P. R. , O'Hara, R. B. , Simpson, G. L. , Solymos, P. , Stevens, M. H. H. , Szoecs, E. , & Wagner, H. (2019). vegan: Community Ecology Package. R package version 2.5‐2. Cran R.

[ece38484-bib-0049] Pernetta, A. P. , Bell, D. J. , & Jones, C. G. (2005). Macro‐ and microhabitat use of Telfair’s skink (*Leiolopisma telfairii*) on Round Island, Mauritius: Implications for their translocation. Acta Oecologica, 28(3), 313–323. 10.1016/j.actao.2005.06.001

[ece38484-bib-0050] Pompanon, F. , Deagle, B. E. , Symondson, W. O. C. , Brown, D. S. , Jarman, S. N. , & Taberlet, P. (2012). Who is eating what: diet assessment using next generation sequencing. Molecular Ecology, 21(8), 1931–1950. 10.1111/j.1365-294X.2011.05403.x 22171763

[ece38484-bib-0051] R Core Team (2021). R: A language and environment for statistical computing. R Foundation for Statistical Computing.

[ece38484-bib-0052] Robeson, M. S. , Khanipov, K. , Golovko, G. , Wisely, S. M. , White, M. D. , Bodenchuck, M. , … Piaggio, A. J. (2018). Assessing the utility of metabarcoding for diet analyses of the omnivorous wild pig (*Sus scrofa*). Ecology and Evolution, 8(1), 185–196. 10.1002/ece3.3638 29321862 PMC5756863

[ece38484-bib-0053] Roswell, M. , Dushoff, J. , & Winfree, R. (2021). A conceptual guide to measuring species diversity. Oikos, 130(3), 321–338. 10.1111/oik.07202

[ece38484-bib-0054] Russo, L. , Memmott, J. , Montoya, D. , Shea, K. , & Buckley, Y. M. (2014). Patterns of introduced species interactions affect multiple aspects of network structure in plant‐pollinator communities. Ecology, 95(10), 2953–2963. 10.1890/13-2229.1

[ece38484-bib-0055] Sax, D. F. , & Gaines, S. D. (2009). Species invasions and extinction: The future of native biodiversity on islands. In the Light of Evolution, 2, 85–106. 10.17226/12501 PMC2556416 18695231

[ece38484-bib-0056] Schlaepfer, M. A. , Sax, D. F. , & Olden, J. D. (2011). The potential conservation value of non‐native species. Conservation Biology, 25(3), 428–437. 10.1111/j.1523-1739.2010.01646.x 21342267

[ece38484-bib-0057] Schloss, P. D. , Westcott, S. L. , Ryabin, T. , Hall, J. R. , Hartmann, M. , Hollister, E. B. , Lesniewski, R. A. , Oakley, B. B. , Parks, D. H. , Robinson, C. J. , Sahl, J. W. , Stres, B. , Thallinger, G. G. , Van Horn, D. J. , & Weber, C. F. (2009). Introducing mothur: Open‐source, platform‐independent, community‐supported software for describing and comparing microbial communities. Applied and Environmental Microbiology, 75(23), 7537–7541. 10.1128/AEM.01541-09 19801464 PMC2786419

[ece38484-bib-0058] Schmidt, J. O. (2009). Defensive behavior. In V. Resh & R. Carde (Eds.), Encyclopedia of Insects, 2nd ed. (pp. 252–257). Academic Press.

[ece38484-bib-0059] Senapathi, D. , Underwood, F. , Black, E. , Nicoll, M. A. C. , & Norris, K. (2010). Evidence for long‐term regional changes in precipitation on the East Coast Mountains in Mauritius. International Journal of Climatology, 30(8), 1164–1177. 10.1002/joc.1953

[ece38484-bib-0060] Silva, L. P. , Mata, V. A. , Lopes, P. B. , Pereira, P. , Jarman, S. N. , Lopes, R. J. , & Beja, P. (2019). Advancing the integration of multi‐marker metabarcoding data in dietary analysis of trophic generalists. Molecular Ecology Resources, 19(6), 1420–1432. 10.1111/1755-0998.13060 31332947 PMC6899665

[ece38484-bib-0061] Strahm, W. A. PhD thesis, University of Reading.

[ece38484-bib-0062] Symondson, W. O. C. (2002). Molecular identification of prey in predator diets. Molecular Ecology, 11(4), 627–641. 10.1046/j.1365-294X.2002.01471.x 11972753

[ece38484-bib-0063] Taberlet, P. , Bonin, A. , Zinger, L. , & Coissac, E. (2018). Environmental DNA. Oxford University Press.

[ece38484-bib-0064] Taberlet, P. , Coissac, E. , Hajibabaei, M. , & Rieseberg, L. H. (2012). Environmental DNA. Molecular Ecology. 10.1111/j.1365-294X.2012.05542.x 22486819

[ece38484-bib-0065] Tercel, M. P. T. G. , Symondson, W. O. C. , & Cuff, J. P. (2021). The problem of omnivory: A synthesis on omnivory and DNA metabarcoding. Molecular Ecology, 30(10), 2199–2206. 10.1111/mec.15903 33772967

[ece38484-bib-0066] Valero‐Mora, P. M. (2010). ggplot2: Elegant graphics for data analysis. Journal of Statistical Software, 35(1), 1–3. 10.18637/jss.v035.b01 21603108

[ece38484-bib-0067] Vinson, J. , & Vinson, J. M. (1969). The Saurian fauna of the Mascarene Islands. The Mauritius Institute Bulletin, 6, 203–320.

[ece38484-bib-0068] Wang, Y. , Naumann, U. , Wright, S. T. , & Warton, D. I. (2012). Mvabund‐ an R package for model‐based analysis of multivariate abundance data. Methods in Ecology and Evolution, 3(3), 471–474. 10.1111/j.2041-210X.2012.00190.x

[ece38484-bib-0069] Zuël, N. (2009). Ecology and Conservation of an Endangered Reptile Community on Round Island, Mauritius. University of Zurich.

