# Supplementary material for: DNA metabarcoding reveals introduced species predominate in the diet of a threatened endemic omnivore, Telfair’s skink (Leiolopisma telfairii)
Source: Ecol Evol. 2021 Dec 21;12(1):e8484. doi: 10.1002/ece3.8484 (PMC8794715; doi:10.1002/ece3.8484)
Supplement: Supplementary file 1 — Supplementary Material [file ECE3-12-e8484-s001.docx]

**Supplementary Information**

S1. *Positive control species composition*

The two positive controls used consisted of a standardised DNA concentration (4 ng / μl) comprised of equal proportions of the following species: *Anthocoris nemorum, Euproctis similis*, *Melieria crassipennis, Metopolophium dirhodum*, *Pardosa palustris*, *Philodromus aureolus*, *Promethes sulcator*, *Sminthurus viridis*, *Tenuiphantes tenuis*, *Tvetenia calvescens,* and *Utomaphora* sp..

S2. *Bioinformatics*

Bioinformatics followed Drake *et al*. (2021) for COI sequencing data (see main text). For ITS2 sequencing data, we followed Moorhouse-Gann *et al*. (in press):

Bioinformatics followed Moorhouse-Gann et al. (Moorhouse-Gann *et al.* in press), including the use of the UniPlant primer pair to amplify ITS2. Following initial bioinformatics steps (refer to Table S1 below and Moorhouse-Gann *et al.* 2021), the blastn algorithm (Altschul 1990) was used in Blast+ (Camacho *et al.* 2009) to assign unique faecal DNA sequences to plant taxa in the ITS2 sequence reference library based on BIT scores. If there was no close match to the local library, sequences were searched against the NCBI Genbank nucleotide database (Benson *et al.* 2018). Custom Python scripts were used to produce a final presence/absence matrix of all plant taxa detected in each sample and reduce the probability of type 1 errors across the dataset (Python scripts available in Moorhouse-Gann *et al*. 2021). Erroneous taxa were removed (e.g. taxa known to be consumed by field teams, fungi, or known contaminants).

Table S1. Results summaries from the initial steps of the bioinformatics pipeline for the floral component of skink diet. The 82 samples in this study were sequenced alongside 903 samples from a related study (to be reported elsewhere) and negatives in two multiplexed pools. All 82 samples were in pool 2. The table below summarises the results from the bioinformatics pipeline for pool 2, prior to the application of the thresholds described above.

| **Bioinformatics process/summary** | **Software** | **Pool 2 summary** |
| --- | --- | --- |
| Raw reads |  | 15,902,919 reads |
| Filter out adapters and low-quality reads (minimum quality score of 20 over a 4 bp sliding window, minimum length of 135 bp) | Trimmomatic v0.32 (Bolger *et al.* 2014) | 11,251,688 reads |
| Align paired reads | Flash v1.2.11 (Magoč and Salzberg 2011) | 8,923,922 reads |
| Extract reads with matching MID-tagged primers (1 bp mismatch allowed) | Mothur v1.37.1 (Schloss *et al.* 2009) | 3,020,763 reads |
| Detection and removal of chimeras, reads with fewer than 10 copies per sample, and reads where the Tracheophyta ITS2 region was undetected | Usearch v9.2.64 (Edgar 2010); ITSx v1.0.11 (Bengtsson-Palme *et al.* 2013) | 1,180,945 reads |
| Summary of read depth per faecal sample |  | Mean reads per sample: 2,411 (range 65 – 12,329) |
| Summary of read depth for negatives and unused MID-tag combinations |  | Mean reads per sample: 384 (range 24 – 3,324) |
| Summary for Round Island faecal samples (total n=246) |  | Mean reads per sample: 3,043 (range: 172 – 12,329) |
| Summary for unique reads originating from Tracheophyta (after removing all duplicate reads) |  | 17,592 unique sequences |
| Summary for unique reads originating from fungi (after removing all duplicate reads) |  | 374 unique sequences |

S3. *Co-occurrence analysis*

To discern whether accidental consumption or secondary predation explained any of the dietary detections, presence-absence data of dietary taxa were used to measure the co-occurrences in each individual Telfair’s skink with package “cooccur” (Griffith *et al.* 2016) to determine positive, random, and negative co-occurrence relationships between all identified dietary taxa. We used the default threshold of the package to remove taxon pairs that did not co-occur in any skink faecal sample. All taxon pairs that co-occurred in at least one skink faecal sample are reported.

Co-occurrence analysis, using presence-absence data, produced taxon pairs that “positively” or “negatively” co-occurred (occurred together significantly more or less, respectively, than expected by chance, *p* = <0.05), as well as randomly co-occurred (did not co-occur together significantly more or less than expected by chance, *p* = ≥0.05). Of the 2,926 taxon pairs measured, 2,715 pairs (92.79%) were removed from the analysis because of insufficient co-occurrences, i.e. those co-occurring fewer times than required for analysis. After these removals, 211 pairs were analysed, resulting in 19 positive, six negative, and 186 random taxon co-occurrences (Figure S1).

Whilst we did find several non-random co-occurrences, only one pair might have explained accidental consumption: a positive co-occurrence relationship between the ant *Brachymyrmex cordemoyi* and the palm *Latania loddigesii*. Because of the way ants forage, we thought it is possible that *B. cordemoyi* swarms over *L. loddigesii* fruits, and this has been observed, but many *B. cordemoyi* detections did not coincide with *L. loddigesii* detections. Furthermore, no other ant species showed this relationship. We also found negative co-occurrence relationships between plants solely or primarily consumed in different seasons. More generally, although co-occurrence analyses can be useful to disentangle results, they are not able to provide strong evidence of ecological interaction in this context (Blanchet *et al.* 2020), and may not facilitate interpretation of results (Tercel *et al.* 2021). This is because there could be a range of other reasons two taxa are co-occurring, which are discussed in detail elsewhere (Blanchet *et al.* 2020).


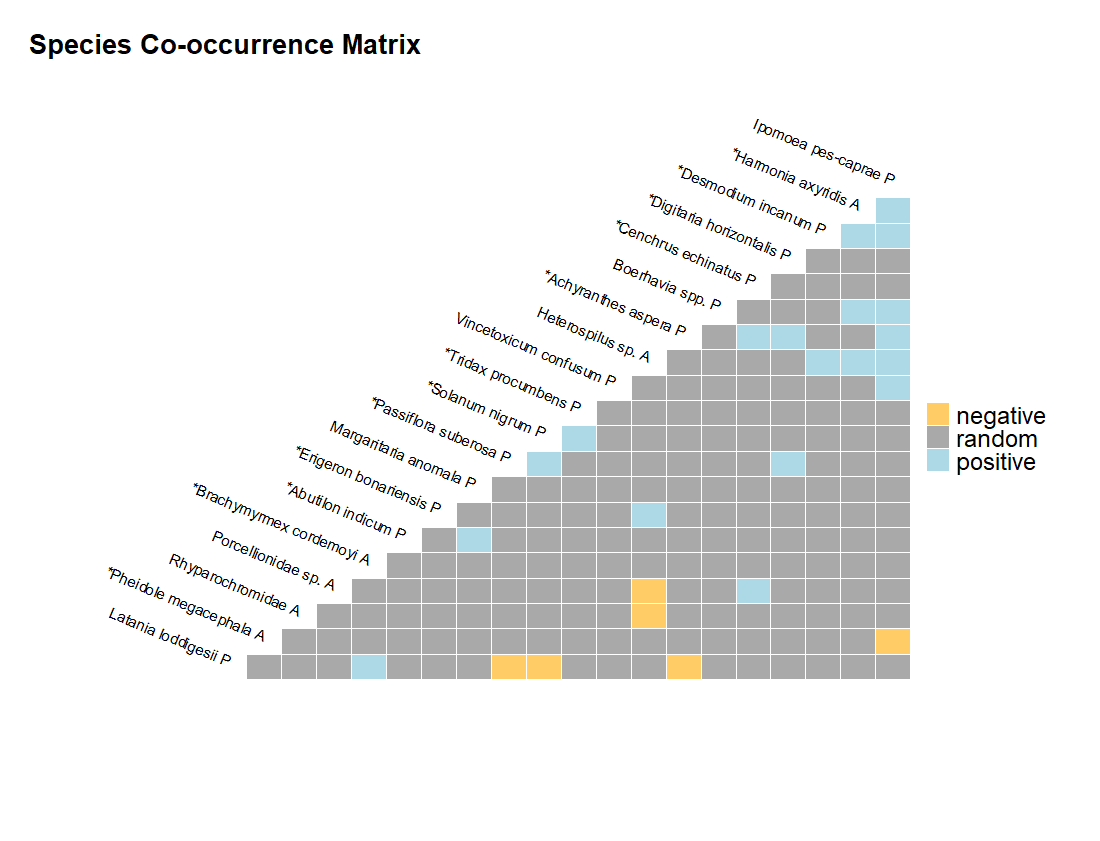


Figure S1. Co-occurrence matrix for species pairs found in Telfair’s skink faecal samples. Yellow squares show species pairs that co-occur significantly less than expected by chance, grey squares show randomly co-occurring species pairs, and light-blue squares show species pairs that co-occur more than expected by chance. * = introduced species; A = animal; P = plant.

Table S2. Taxonomic information, status relative to Round Island (introduced, cryptogenic, native, endemic), number of detections, and frequency of occurrence (%) of all dietary items found in Telfair’s skink faecal samples after data clean-up (n = 73).

| Kingdom | Phylum | Class | Order | Family | Dietary taxon | Status | Detections | *F*_o_ (%) |
| --- | --- | --- | --- | --- | --- | --- | --- | --- |
| Animal | Arthropoda | Arachnida | Araneae | Theridiidae | *Coleosoma floridanum* | introduced | 1 | 1.37 |
|  |  |  |  | Theridiidae | Theridiidae sp. | cryptogenic | 1 | 1.37 |
|  |  |  | Araneae | Thomisidae | *Ozyptila claveata* | introduced | 4 | 5.48 |
|  |  |  | Mesostigmata | Laelapidae | Laelapidae sp. | cryptogenic | 1 | 1.37 |
|  |  |  | Opiliones | Phalangodidae | *Bishopella laciniosa* | introduced | 1 | 1.37 |
|  |  |  | Sarcoptiformes | Scheloribatidae | Scheloribatidae sp. | cryptogenic | 1 | 1.37 |
|  |  | Collembola | Entomobryomorpha | Entomobryidae | Entomobryidae sp. | cryptogenic | 1 | 1.37 |
|  |  | Crustacea | Decapoda | Grapsidae | *Geograpsus grayi* | native | 1 | 1.37 |
|  |  |  | Isopoda | Porcellionidae | Porcellionidae sp. | cryptogenic | 25 | 34.25 |
|  |  | Insecta | Blattodea | Blaberidae | Blaberidae sp. | cryptogenic | 2 | 2.74 |
|  |  |  | Coleoptera | Coccinellidae | *Harmonia yedoensis* | introduced | 15 | 20.55 |
|  |  |  | Diptera | Drosophilidae | *Drosophila* sp. | cryptogenic | 2 | 2.74 |
|  |  |  |  | Drosophilidae | *Zaprionus africanus* | cryptogenic | 1 | 1.37 |
|  |  |  |  | Drosophilidae | *Zaprionus indianus* | introduced | 4 | 5.48 |
|  |  |  |  | Tachinidae | *Chetogena* sp. | cryptogenic | 2 | 2.74 |
|  |  |  |  | Tephritidae | *Ceratitis capitata* | introduced | 1 | 1.37 |
|  |  |  | Embioptera | Oligotomidae | *Oligotoma saundersii* | introduced | 2 | 2.74 |
|  |  |  | Hemiptera | Aleyrodidae | *Dialeurodes hongkongensis* | introduced | 2 | 2.74 |
|  |  |  |  | Cydnidae | Cydnidae sp. | native | 1 | 1.37 |
|  |  |  |  | Diaspididae | *Hemiberlesia lataniae* | native | 1 | 1.37 |
|  |  |  |  | Pseudococcidae | *Planococcus minor* | introduced | 1 | 1.37 |
|  |  |  |  | Rhyparochromidae | Rhyparochromidae sp. | cryptogenic | 6 | 8.22 |
|  |  |  | Hymenoptera | Apidae | *Inquilina* sp. | native | 2 | 2.74 |
|  |  |  |  | Braconidae | *Heterospilus* sp. | cryptogenic | 29 | 39.73 |
|  |  |  |  | Chalcididae | *Brachymeria* sp. | cryptogenic | 1 | 1.37 |
|  |  |  |  | Formicidae | *Brachymyrmex cordemoyi* | introduced | 14 | 19.18 |
|  |  |  |  | Formicidae | Formicidae sp. | cryptogenic | 2 | 2.74 |
|  |  |  |  | Formicidae | *Monomorium floricola* | introduced | 2 | 2.74 |
|  |  |  |  | Formicidae | *Pheidole megacephala* | introduced | 29 | 39.73 |
|  |  |  |  | Formicidae | *Tapinoma* sp. | introduced | 1 | 1.37 |
|  |  |  |  | Formicidae | *Tetramorium simillimum* | introduced | 1 | 1.37 |
|  |  |  |  | Hymenoptera | Hymenoptera sp. | cryptogenic | 1 | 1.37 |
|  |  |  |  | Platygastridae | Platygastridae sp. | cryptogenic | 2 | 2.74 |
|  |  |  | Lepidoptera | Crambidae | Crambidae sp. | native | 1 | 1.37 |
|  |  |  |  | Geometridae | Geometridae sp. | native | 1 | 1.37 |
|  |  |  | Orthoptera | Gryllidae | Gryllidae sp. | native | 1 | 1.37 |
|  |  |  | Thysanoptera | Thripidae | *Thrips parvispinus* | introduced | 1 | 1.37 |
|  | Chordata | Aves | Procellariiformes | Procellariidae | *Ardenna pacifica* | native | 1 | 1.37 |
|  | Mollusca | Gastropoda | Stylommatophora | Succineidae | *Succinea manuana* | introduced | 1 | 1.37 |
| Plant | Angiosperms | Eudicots | Asterales | Asteraceae | *Bidens pilosa* | introduced | 1 | 1.37 |
|  |  |  |  |  | *Erigeron bonariensis* | introduced | 4 | 5.48 |
|  |  |  |  |  | *Psiadia arguta* | endemic | 1 | 1.37 |
|  |  |  |  |  | *Tridax procumbens* | introduced | 8 | 10.96 |
|  |  |  |  | Goodeniaceae | *Scaevola taccada* | native | 8 | 10.96 |
|  |  |  | Caryophyllales | Amaranthaceae | *Achyranthes aspera* | introduced | 14 | 19.18 |
|  |  |  |  |  | *Amaranthus viridis* | introduced | 2 | 2.74 |
|  |  |  |  | Nyctaginaceae | *Boerhavia* sp. | native | 14 | 19.18 |
|  |  |  |  | Portulacaceae | *Portulaca oleracea* | introduced | 1 | 1.37 |
|  |  |  | Celastrales | Celastraceae | *Elaeodendron orientale* | endemic | 1 | 1.37 |
|  |  |  |  |  | *Maytenus pyria* | endemic | 2 | 2.74 |
|  |  |  | Fabales | Fabaceae | *Desmanthus virgatus* | introduced | 1 | 1.37 |
|  |  |  |  |  | *Desmodium incanum* | introduced | 5 | 6.85 |
|  |  |  |  |  | *Gagnebina pterocarpa* | native | 8 | 10.96 |
|  |  |  | Gentianales | Apocynaceae | *Vincetoxicum confusum* | native | 9 | 12.33 |
|  |  |  |  | Rubiaceae | *Morinda citrifolia* | introduced | 1 | 1.37 |
|  |  |  | Lamiales | Lamiaceae | *Volkameria heterophylla* | endemic | 1 | 1.37 |
|  |  |  |  |  | *Premna serratifolia* | native | 4 | 5.48 |
|  |  |  | Malpighiales | Euphorbiaceae | *Euphorbia thymifolia* | cryptogenic | 3 | 4.11 |
|  |  |  |  | Passifloraceae | *Passiflora suberosa* | introduced | 11 | 15.07 |
|  |  |  |  |  | *Turnera angustifolia* | introduced | 1 | 1.37 |
|  |  |  |  | Phyllanthaceae | *Margaritaria anomala* | endemic | 3 | 4.11 |
|  |  |  | Malvales | Malvaceae | *Abutilon indicum* | introduced | 26 | 35.62 |
|  |  |  |  |  | *Hibiscus tiliaceus* | native | 4 | 5.48 |
|  |  |  |  |  | *Hilsenbergia petiolaris* | native | 3 | 4.11 |
|  |  |  |  |  | *Thespesia populnea* | native | 1 | 1.37 |
|  |  |  | Myrtales | Myrtaceae | *Eugenia lucida* | endemic | 3 | 4.11 |
|  |  |  | Sapindales | Meliaceae | *Turraea thouarsiana* | endemic | 1 | 1.37 |
|  |  |  |  | Sapindaceae | *Dodonaea viscosa* | native | 1 | 1.37 |
|  |  |  | Solanales | Convolvulaceae | *Ipomoea pes-caprae* | native | 16 | 21.92 |
|  |  |  |  | Solanaceae | *Solanum lycopersicum* | introduced | 3 | 4.11 |
|  |  |  |  |  | *Solanum nigrum* | introduced | 13 | 17.81 |
|  |  | Monocots | Arecales | Arecaceae | *Hyophorbe lagenicaulis* | endemic | 1 | 1.37 |
|  |  |  |  |  | *Latania loddigesii* | endemic | 24 | 32.88 |
|  |  |  | Poales | Poaceae | *Cenchrus echinatus* | introduced | 10 | 13.70 |
|  |  |  |  |  | *Chloris barbata* | introduced | 2 | 2.74 |
|  |  |  |  |  | *Dactyloctenium ctenoides* | native | 3 | 1.37 |
|  |  |  |  |  | *Digitaria horizontalis* | introduced | 9 | 1.37 |

| Status | Kingdom | Dietary taxon richness | Total detections | Mean detections (± SE) |
| --- | --- | --- | --- | --- |
| Introduced | Animals | 16 | 80 | 5 (± 1.95) |
|  | Plants | 17 | 112 | 6.59 (± 1.64) |
|  | **Total** | **33** | **192** | **5.82 (± 1.24)** |
| Cryptogenic | Animals | 15 | 77 | 5.13 (± 2.32) |
|  | Plants | 1 | 3 | 3 |
|  | **Total** | **16** | **80** | **5 (± 2.11)** |
| Native | Animals | 8 | 9 | 1.13 (± 0.13) |
|  | Plants | 11 | 71 | 6.45 (± 1.52) |
|  | **Total** | **19** | **80** | **4.21 (± 1.04)** |
| Endemic | Animals | 0 | 0 | 0 |
|  | Plants | 10 | 37 | 4.11 (± 2.37) |
|  | **Total** | **9** | **37** | **4.11 (± 2.36)** |
| **Total** |  | **77** | **389** | **5.05 (± 0.79)** |

Table S3. Dietary taxon richness, total dietary taxon detections, and mean (± SE) dietary taxon detections of Telfair’s skinks by dietary taxon status relative to Round Island (cryptogenic, endemic, introduced, or native) and taxonomic kingdom. Mean dietary taxon detections per taxon within each category were calculated by dividing total detections by the number of dietary taxa detected.

References

Altschul, S. (1990). Basic Local Alignment Search Tool. *Journal of Molecular Biology*. doi: https://doi.org/10.1006/jmbi.1990.9999.

Bengtsson-Palme, J., Ryberg, M., Hartmann, M., Branco, S., Wang, Z., Godhe, A., … Nilsson, R.H. (2013). Improved software detection and extraction of ITS1 and ITS2 from ribosomal ITS sequences of fungi and other eukaryotes for analysis of environmental sequencing data. *Methods in Ecology and Evolution*. doi: https://doi.org/10.1111/2041-210X.12073.

Benson, D.A., Cavanaugh, M., Clark, K., Karsch-Mizrachi, I., Ostell, J., Pruitt, K.D. and Sayers, E.W. (2018). GenBank. *Nucleic Acids Research* **46**(D1):D41–D47. doi: https://doi.org/10.1093/nar/gkx1094.

Blanchet, F.G., Cazelles, K. and Gravel, D. (2020). Co-occurrence is not evidence of ecological interactions. *Ecology Letters*. doi: https://doi.org/10.1111/ele.13525.

Bolger, A.M., Lohse, M. and Usadel, B. (2014). Trimmomatic: A flexible trimmer for Illumina sequence data. *Bioinformatics*. doi: https://doi.org/10.1093/bioinformatics/btu170.

Camacho, C., Coulouris, G., Avagyan, V., Ma, N., Papadopoulos, J., Bealer, K. and Madden, T.L. (2009). BLAST+: Architecture and applications. *BMC Bioinformatics*. doi: https://doi.org/10.1186/1471-2105-10-421.

Drake, L.E., Cuff, J.P., Young, R.E., Marchbank, A., Chadwick, E.A. and Symondson, W.O.C. (2021). An assessment of minimum sequence copy thresholds for identifying and reducing the prevalence of artefacts in dietary metabarcoding data. *Methods in Ecology and Evolution*. doi: https://doi.org/10.1111/2041-210X.13780.

Edgar, R.C. (2010). Search and clustering orders of magnitude faster than BLAST. *Bioinformatics*. doi: https://doi.org/10.1093/bioinformatics/btq461.

Griffith, D.M., Veech, J.A. and Marsh, C.J. (2016). Cooccur: Probabilistic species co-occurrence analysis in R. *Journal of Statistical Software* **69**(1):1–17. doi: https://doi.org/10.18637/jss.v069.c02.

Magoč, T. and Salzberg, S.L. (2011). FLASH: Fast length adjustment of short reads to improve genome assemblies. *Bioinformatics*. doi: https://doi.org/10.1093/bioinformatics/btr507.

Moorhouse-Gann, R., Vaughan, I.P., Cole, N., Goder, M., Tatayah, V., Jones, C., … Symondson, W.O.C. Impacts of Ecological Replacement on an Island Ecosystem. *Journal of Applied Ecology*. doi: https://doi.org/10.1111/1365-2664.14096.

Schloss, P.D., Westcott, S.L., Ryabin, T., Hall, J.R., Hartmann, M., Hollister, E.B., … Weber, C.F. (2009). Introducing mothur: Open-source, platform-independent, community-supported software for describing and comparing microbial communities. *Applied and Environmental Microbiology*. doi: https://doi.org/10.1128/AEM.01541-09.

Tercel, M.P.T.G., Symondson, W.O.C. and Cuff, J.P. (2021). The problem of omnivory: A synthesis on omnivory and DNA metabarcoding. *Molecular Ecology* **30**(10):2199–2206. doi: https://doi.org/10.1111/mec.15903.
